# Mitochondrial Calcium Uniporter‐Mediated Regulation of the SIRT3/GSK3β/β‐Catenin Signaling Pathway in Vascular Remodeling

**DOI:** 10.1096/fj.202500369RR

**Published:** 2025-07-07

**Authors:** Min Jiang, Lejian Lin, Shuai Yue, Shujin Shi, Haojie Yan, Hui Yi, Fan Han, Shuai Xu, Junjie Su, Ran Zhang

**Affiliations:** ^1^ Department of Pulmonary and Critical Care Medicine Chinese PLA General Hospital Beijing China; ^2^ Department of Cardiovascular Medicine Chinese PLA General Hospital Beijing China; ^3^ Graduate School of Chinese PLA Medical School Beijing China; ^4^ Division of Pediatric Cardiology, Department of Pediatrics The Seventh Medical Center of Chinese PLA General Hospital Beijing China

**Keywords:** angiotensin II, calcium, GSK3β/β‐catenin, hindlimb unloading, microgravity, mitochondrial calcium uniporter, SIRT3, vascular remodeling

## Abstract

Calcium homeostasis plays a crucial role in regulating the phenotype of vascular smooth muscle cells (VSMCs) and vascular remodeling. This study aims to investigate the role of the mitochondrial calcium uniporter (MCU), which facilitates the uptake of Ca^2+^ into the mitochondria, in vascular remodeling and its underlying regulatory mechanisms. Vascular remodeling in rats was induced through either 21‐day hindlimb unloading (HU) or 21‐day angiotensin II (Ang II) infusion (0.7 mg/kg/day). Phenotypic switching of VSMCs and vascular remodeling were assessed. To induce phenotypic switching and clarify the underlying regulatory mechanisms, VSMCs were treated with Ang II (100 μmol/L). Gene manipulation was performed using plasmids, lentivirus, and adeno‐associated virus serotype 9 (AAV9). Mitochondrial oxidative stress, Ca^2+^ distribution, and the expression of MCU, SIRT3, GSK3β, and β‐catenin, along with GSK3β activity, SIRT3 ubiquitination, and GSK3β acetylation, were evaluated. The expression of MCU and SIRT3 in rat cerebral arteries was downregulated following HU and Ang II administration, which resulted in an increase in cytoplasmic Ca^2+^, a decrease in mitochondrial Ca^2+^, and a shift toward a synthetic phenotype in VSMCs. In vitro, Ang II treatment of VSMCs led to reduced expression of MCU, SIRT3, and GSK3β, and increased nuclear translocation of β‐catenin. Knockdown of MCU caused an increase in cytoplasmic Ca^2+^ and a reduction in mitochondrial Ca^2+^, while MCU overexpression had the opposite effect, decreasing cytoplasmic Ca^2+^ and increasing mitochondrial Ca^2+^. Additionally, MCU overexpression decreased SIRT3 ubiquitination, mitochondrial oxidative stress, GSK3β acetylation, nuclear translocation of β‐catenin, and VSMC phenotypic switching—these effects were blocked by SIRT3 knockdown. Moreover, MCU overexpression partially mitigated vascular remodeling in HU and hypertensive rats by inhibiting the GSK3β/β‐catenin pathway and preserving SIRT3. Ang II regulates MCU protein expression, which is reduced in the HU and Ang II‐induced hypertensive rat cerebral arteries. This reduction impairs cellular Ca^2+^ buffering and promotes mitochondrial oxidative stress. The stress response triggers the downstream degradation of SIRT3, which subsequently inhibits the activity of GSK3β via acetylation and promotes the nuclear translocation of β‐catenin, thereby facilitating phenotypic switching and vascular remodeling.

## Graphical abstract

In HU and hypertensive rats, MCU expression in cerebral arteries is reduced. This reduction impairs cellular Ca^2+^ buffering and promotes mitochondrial oxidative stress. The stress response initiates the downstream degradation of SIRT3, which subsequently inhibits the activity of GSK3β via acetylation and promotes the nuclear translocation of β‐catenin, thereby facilitating phenotypic switching and vascular remodeling.

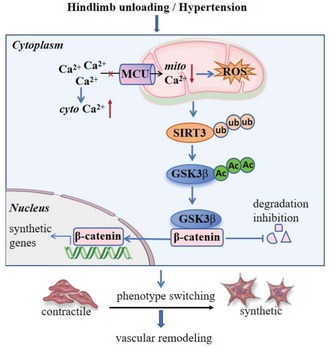

## 1 Introduction

Exposure to microgravity results in post‐flight cardiovascular dysfunction in astronauts, which compromises both spaceflight safety and cardiovascular health [1]. Region‐specific vascular remodeling plays a key role in this process, with the cerebral arteries undergoing hypertrophy and enhanced vasoreactivity due to the net migration of fluid from the lower to the upper body [2]. The alterations in cerebrovascular structure and function under microgravity resembled those seen in hypertension [3], possibly due to the similar cerebrovascular stress in both conditions and subsequent hyperactivation of the renin‐angiotensin system (RAS) [4]. We have reported that synthetic phenotypic switching, proliferation of vascular smooth muscle cells (VSMCs), and cerebrovascular remodeling occur via the PERK‐eIF2α‐ATF4‐CHOP and ERα‐NRF1‐OMI‐mitophagy signaling pathways in ground‐based simulations of microgravity [5, 6]. Despite significant progress in this field, the underlying mechanisms remain to be fully elucidated.

Calcium homeostasis plays a crucial role in both physiological and pathological vascular remodeling. Ca^2+^ plays a multifaceted role in regulating the function of VSMCs, including vascular tone, cell proliferation, apoptosis, and phenotypic switching. We previously found that mitochondria regulate calcium homeostasis, with elevated cytoplasmic Ca^2+^ levels and decreased mitochondrial Ca^2+^ levels in cerebrovascular VSMCs of hindlimb unloading (HU) rats [7]. The mitochondrial calcium uniporter (MCU) on the inner mitochondrial membrane is responsible for the uptake of Ca^2+^ into the mitochondria. The MCU exhibits Ca^2+^ selectivity at low cytoplasmic Ca^2+^ concentrations, enabling the dynamic uptake and release of Ca^2+^ to buffer cytoplasmic Ca^2+^ levels. Despite the well‐established effects of MCU in Ca^2+^ signaling, its involvement in vascular remodeling remains controversial. MCU‐dependent mitochondrial calcium‐induced mitophagy contributes to the proliferation of VSMCs in response to apelin‐13 stimulation [8]. However, MCU is diminished in VSMCs in pulmonary arterial hypertension, while upregulation of MCU reduces cell migration, proliferation, and resistance to apoptosis by increasing cytoplasmic Ca^2+^ but decreasing mitochondrial Ca^2+^ in pulmonary artery smooth muscle cells [9]. A recent study indicates that MCU is regulated by angiotensin II (Ang II) in cardiomyocytes [10], but there is no evidence to suggest this regulation occurs in VSMCs. Notably, the role of MCU in vascular remodeling in the context of Ang II‐induced hypertension and microgravity, as well as its impact on VSMC function, remains to be elucidated.

SIRT3 is a mitochondrial deacetylase that helps maintain metabolic homeostasis under various conditions by deacetylating multiple enzymes. It can deacetylate glycogen synthase kinase‐3 beta (GSK3β), a key protein kinase involved in various cellular functions and diseases, including VSMC calcification [11] and Marfan syndrome [12]. Despite these findings, the interaction between SIRT3 and GSK3β during phenotypic switching of VSMCs in microgravity remains poorly understood. In this study, we employed gene manipulation models to investigate the role of MCU in vascular remodeling and the underlying mechanisms driving this process. We specifically focused on how MCU regulates the SIRT3‐GSK3β deacetylation pathway and its role in influencing the phenotypic switching of cerebral VSMCs in vascular remodeling.

## 2 Materials and Methods

The experimental procedure and treatment of rats were in accordance with the Guiding Principles for the Care and Use of Animals in the Field of Physiological Sciences and complied with Chinese guidelines for experimental animals (MDL2024‐01‐06‐01).

### 2.1 Animal and Tissue Preparation

For the ground‐based simulation of microgravity, male Sprague–Dawley rats were randomly assigned to one of eight groups: control (CON), hindlimb unloading (HU), CON+Losartan, HU+Losartan, CON+AAV9‐SM22a‐NC, CON+AAV9‐SM22a‐MCU, HU+AAV9‐SM22a‐NC, HU+AAV9‐SM22a‐MCU. The technique for hindlimb unloading has been previously described in detail [5, 6]. Briefly, the rats were suspended by their tails, with their hindlimbs positioned 1 cm above the cage floor while their forelimbs were allowed to touch the ground. This resulted in the body forming a 30° angle with the ground.

For Ang II‐induced hypertension, male Sprague–Dawley rats were randomly assigned to eight groups: control (CON), hypertension (HTN), CON+Losartan, HTN+Losartan, CON+AAV9‐SM22a‐NC, CON+AAV9‐SM22a‐MCU, HTN+AAV9‐SM22a‐NC, HTN+AAV9‐SM22a‐MCU. To prepare the Ang II and the pump, we begin by calculating the total amount of Ang II required based on the animal's weight. For example, a 200 g rat would need approximately 2.94 mg of Ang II for a 21‐day period. A 15 mg/mL Ang II solution is then prepared using sterile saline and filtered to remove any bacterial contaminants. Finally, the solution is loaded into a 200 μL Alzet micro‐pump. After 1 week of ad libitum administration of 1% NaCl and 0.01% N‐nitro L‐arginine methyl ester in the drinking water, the pump was surgically implanted in the subdermal dorsal area to continuously deliver Ang II at a rate of 0.7 mg/kg/day. The CON group underwent the same procedure but without pump implantation. Animals were acclimatized to blood pressure and heart rate measurements for three consecutive weeks (once per week). Following this, blood pressure and heart rate were recorded at baseline and at the end of each experimental week using a non‐invasive tail‐cuff photoplethysmographic method (Zhongshi Technology, Beijing, China).

For Losartan administration, the HU+Losartan, Ang II+Losartan, and Con+Losartan groups received distilled water containing losartan (#HY‐17512, MedChemExpress, NJ, USA) at a dose of 30 mg/kg/day via gavage, while rats in the other groups were given an equal volume of vehicle (distilled water). On day 1, as preparations for the HU and hypertensive rats began, losartan administration was initiated immediately.

For vascular‐specific MCU gene transfection, AAV9‐SM22a‐MCU (1 × 10^12^ vg/rat) or AAV9‐SM22a‐NC (1 × 10^12^ vg/rat) was injected via the tail vein on day 7 of modeling.

All rats were provided with standard laboratory chow and water ad libitum and were housed individually in a room maintained on a 12‐h light/dark cycle.

After 21 days of modeling, the rats were anesthetized with 5% isoflurane and euthanized by exsanguination via the abdominal aorta. The cerebral arteries were promptly transferred to ice‐cold phosphate‐buffered saline (PBS), then frozen in liquid nitrogen and stored at −80°C for protein and mRNA analysis. The basilar arteries were rapidly fixed in 4% paraformaldehyde, embedded in paraffin, and sectioned for hematoxylin and eosin staining, as well as immunohistochemistry.

### 2.2 Cell Culture

A7r5 cells (rat thoracic aortic smooth muscle cells) were purchased from Zhongqiao Xinzhou Biotechnology Co. Ltd. (ZQ0139, Shanghai, China). The cells were cultured in DMEM (Hyclone, USA) containing 10% fetal bovine serum (FBS) (Gibco, MA, USA) and 1% penicillin/streptomycin (Gibco, MA, USA). The cells were cultured at 37°C with 5% CO_2_.

### 2.3 siRNA Transfection

Small interfering RNAs (siRNAs) targeting rat MCU and SIRT3, along with their non‐targeting sequences (negative controls), were designed and synthesized by Reagent Biological Technology (Reagent, Beijing, China). Rat VSMCs were washed and incubated with 20 nM siRNA in OptiMEM media (#244496, Gibco, CA, USA) supplemented with 1:40 Lipofectamine 3000 reagent (#2343152, Thermo Fisher, Waltham, MA) for 6 h. Cells were washed with PBS and were then incubated overnight with complete DMEM medium with 10% FBS. 48 h later, cells were collected for experimentation. The sequences were listed in Table 1.

### 2.4 SIRT3 and MCU Overexpression

The pGMLV‐CMV‐SIRT3 and pGMLV‐CMV‐MCU plasmids were designed and produced by the Reagent Biological Technology Co. Ltd. (Reagent, Beijing, China) and subsequently transfected into 293 T cells using Lenti‐HG Mix (#Gmeasy, Genomeditech, Shanghai, China). After transfection for 48 h, the viral supernatant was collected and filtered through a 0.22‐μm filter to obtain lentivirus‐MCU (LV‐MCU) and lentivirus‐SIRT3 (LV‐SIRT3).

To achieve VSMC‐specific MCU overexpression in vivo, the GPAAV‐SM22a‐MCU‐T2A‐eGFP‐WPRE plasmid was designed and produced by Reagent Biological Technology Co. Ltd. and transfected into AAV Pro‐293T (#632273, Takara, Beijing, China) using HG Transgene Reagent (TG‐10012, Genomeditech, Shanghai, China). After 8 h of transfection, enhancing buffer was added (Genomeditech, Shanghai, China). After transfection for 72 h, viral supernatant was collected and filtered through a 0.22‐μm filter to obtain AAV9‐SM22a‐MCU. The sequences were listed in Table 1.

### 2.5 RT‐PCR

Total RNA was extracted from tissues using TRNzol reagent (#DP424, TIANGEN, Beijing, China). Following the manufacturer's instructions, 500 ng of RNA was reverse transcribed using the RevertAid First Strand cDNA Synthesis Kit (#K1622, Thermo, Waltham, MA, USA). Quantitative real‐time PCR was performed using SYBR Green qPCR Master Mix (#B21203, Selleck, Houston, TX, USA) and the Applied Biosystems 7500 Real‐Time PCR System (ABI, Carlsbad, CA, USA). GAPDH was used as the internal reference for quantifying mRNA levels. The primers are as listed in Table 1.

**TABLE 1:** The sequences of the primers, oligonucleotides, and plasmids used in the study.

Name	Sequence (5′–3′)
MYH11‐Forward	CATCATCCACTATGCTGGGAAGGT
MYH11‐Reverse	AGGTCACATTATCATTTAGCGGGTC
SPP1‐Forward	GTGGTTTGCTTTTGCCTGTTC
SPP1‐Reverse	AAGATTCTGCTTCTGAGATGGGTC
GAPDH‐Forward	TTCAGCTCTGGGATGACCTT
GAPDH‐Reverse	TGCCACTCAGAAGACTGTGG
LV‐MCU‐Forward	GCGAATTCGAAGTATACCTCGAGGC
LV‐MCU‐Reverse	CATGGTCTTTGTAGTCGGATCCATC
si‐MCU‐1‐Forward	GCUGUCAGUUCACACUCAATT
si‐MCU‐1‐Reverse	UUGAGUGUGAACUGACAGCTT
si‐MCU‐2‐Forward	GCUGGUCAUUAAUGACUUATT
si‐MCU‐2‐Reverse	UAAGUCAUUAAUGACCAGCTT
si‐MCU‐3‐Forward	GGAGCUUAUUGAAAGACUATT
si‐MCU‐3‐Reverse	UAGUCUUUCAAUAAGCUCCTT
LV‐SIRT3‐Forward	CGCTATGTGGATACGCTGCTTTA
LV‐SIRT3‐Reverse	GCAACCAGGATTTATACAAGGAGGA
si‐SIRT3‐1‐Forward	CACUUCGCUUUGGCUUGAATT
si‐SIRT3‐1‐Reverse	UUCAAGCCAAAGCGAAGUGTT
si‐SIRT3‐2‐Forward	GAGUCCUGCCAAGCCCAGATT
si‐SIRT3‐2‐Reverse	UCUGGGCUUGGCAGGACUCTT
si‐SIRT3‐3‐Forward	CUUGGCCUUGCGUGCUCAUTT
si‐SIRT3‐3‐Reverse	AUGAGCACGCAAGGCCAAGTT
AAV9‐SM22a‐MCU‐Forward	CGCGAATTCGAAGTATACCTCGAG
AAV9‐SM22a‐MCU‐Reverse	ACTTCCTCTGCCCTCGGATCC

### 2.6 Western Blot

Western blot analysis was performed as previously described [13]. Briefly, total proteins extracted from cells and tissues were separated by 8% to 12% SDS‐PAGE and transferred to nitrocellulose or PVDF membranes. The membranes were then blocked with 5% milk or BSA in TBST. After incubation with the primary antibody at 4°C overnight, the membranes were incubated with horseradish peroxidase‐conjugated anti‐mouse or anti‐rabbit antibodies for 2 h. The blots were washed and developed with an enhanced chemiluminescent system (Amersham Biosciences, Uppsala, Sweden). The primary antibodies used were GAPDH (#60004‐1‐Ig), GSK3β (#22104‐1‐AP), SIRT3 (#10099‐1‐AP), β‐catenin (#51967‐1‐AP), α‐SMA (#14395‐1‐AP), OPN (#30200‐1‐AP), and PCNA (#60097‐1‐Ig) all purchased from Proteintech (Wuhan, China). MCU (#bs‐20189R) was purchased from Bioss (Bioss, MA, USA). Ace‐K (#A1525) and ubiquitin (#A0162) were purchased from ABclonal Technology Co. Ltd. (ABclonal, Wuhan, China). p‐GSK‐3β Ser9 (#5558) was purchased from Cell Signaling Technology (Danvers, MA, USA).

### 2.7 Cytoplasmic and Mitochondrial Ca^2+^ Assay

Cytosolic and mitochondrial Ca^2+^ concentrations were measured using the Ca^2+^ indicators Fluo‐4 AM (#S1060, Beyotime, Beijing, China) and Rhod‐2 AM (#S1062, Beyotime, Beijing, China) with a flow cytometry system, respectively. The VSMCs were incubated in Hank's balanced salt solution (Sigma‐Aldrich, St. Louis, MO) containing 5 μmol/L Fluo‐4 AM and 5 μmol/L Rhod‐2 AM at 37°C for 60 min. The Ca^2+^ content in individual VSMC groups was analyzed using the BD LSRFortessa flow cytometer (BD Biosciences, Franklin Lakes, NJ, USA).

### 2.8 Nuclear Fractionation

The nuclear and cytoplasmic extracts from VSMCs were prepared with a Nuclear Protein Extraction Kit (#P0027, Beyotime, Beijing, China) supplemented with a protease inhibitor cocktail (#ST507, Beyotime, Beijing, China).

### 2.9 Immunoprecipitation

VSMCs (1 × 10^6^) were lysed in NETN buffer (20 mM Tris–HCl [pH = 8.0], 150 mM NaCl, 2 mM EDTA, and 0.5% NP‐40), supplemented with a proteinase inhibitor cocktail. A total of 800 μg of the lysate was incubated overnight at 4°C with either anti‐SIRT3 antibody (2 μg, #A20805, Abclonal, Wuhan, China), anti‐GSK3β antibody (2 μg, #A2081, Abclonal, Wuhan, China), or control IgG (2 μg). Following incubation, 50 μL of protein A/G agarose (Santa Cruz, CA, USA) was added, and the mixture was incubated for an additional 3 h. The precipitates were washed three times with NETN buffer and then resuspended in 2× SDS‐PAGE sample buffer. After boiling at 95°C for 10 min, protein blot analysis was performed.

### 2.10 GSK3β Activity Assay

A GSK3β activity assay was performed using a commercially available GSK3β activity assay kit (#CS0990, Merck, Kenilworth, NJ, USA) according to the manufacturer's protocol.

### 2.11 Mitochondrial ROS Assay

MitoSOX Red Mitochondrial Superoxide Indicator (#S0061S, Beyotime, Beijing, China) was used to stain mitochondrial reactive oxidative species (mtROS). Briefly, cells were incubated with MitoSOX Red for 30 min at 37°C with 5% CO_2_ in the dark. The samples were then washed with PBS to remove excess probe. Nuclei were stained with DAPI. mtROS quantification was based on the fluorescence intensity of mtROS.

### 2.12 Immunohistochemistry

Paraffin‐embedded sections of the basilar artery (3 μm) were mounted on Thermo Scientific Superfrost Plus slides and allowed to dry overnight. The arterial sections were incubated with primary antibodies against OPN, PCNA, and α‐SMA (Proteintech, Wuhan, China). After washing with PBS, the sections were developed using 3,3′‐diaminobenzidine (Roche Diagnostics, Mannheim, Germany) as the substrate for horseradish peroxidase. The sections were then rinsed, dehydrated with ethanol, cleared with xylene, and mounted. OPN, PCNA, and α‐SMA staining of basilar artery sections were observed under a microscope. In each group, basilar arteries were harvested from 5 rats, and 3 sections were measured for each vascular specimen. Each section was evaluated at 0°, 90°, 180°, and 270° around the arterial ring. The 12 measurements were then averaged to determine the wall thickness.

### 2.13 Statistical Analysis

All quantitative data are presented as mean ± SEM. The comparison between two groups was conducted using a Student's *t‐*test for statistical evaluation. For comparisons involving more than two groups, one‐way analysis of variance (ANOVA) was used and Dunnett test was used as post hoc test. For the comparisons of blood pressure, two‐way ANOVA and Tukey's multiple comparison test were used. All statistical analyses were performed using GraphPad Prism 8.0. Differences were considered significant at *p < 0.05, **p < 0.01, ***p < 0.001, and ****p < 0.0001.

## 3 Results

### 3.1 The Effects of HU and Ang II on MCU, SIRT3, GSK3β Expression, and Calcium Homeostasis in Cerebrovascular VSMCs

**FIGURE 1:**
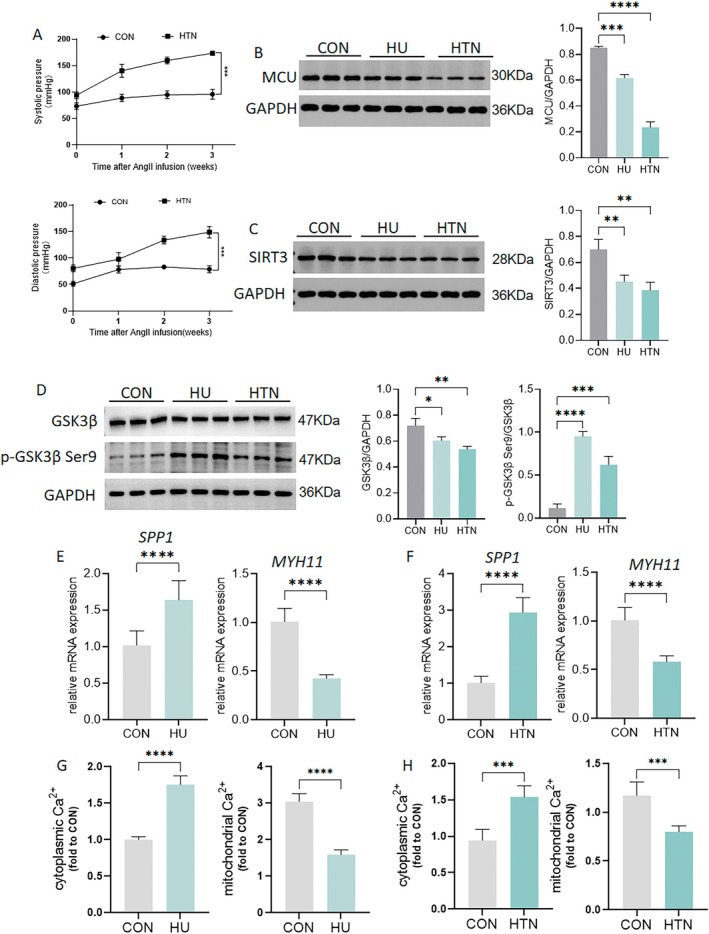
Expression of MCU, SIRT3, and GSK3β, calcium homeostasis and phenotypic markers in cerebral arteries of rats with hindlimb unloading (HU) and angiotensin II (Ang II)‐induced hypertension. Rats were subjected to 21‐day HU to simulate microgravity effects and treated with a 21‐day Ang II infusion (0.7 mg/kg/day) to establish hypertension. (A) Noninvasive tail cuff monitoring of systolic blood pressure (SBP) and diastolic blood pressure (DBP) in rats with vehicle or Ang II for 21 days. (B–D) Western blot analyses of MCU, SIRT3, GSK3β and p‐GSK3β Ser9 in HU and hypertensive (HTN) rats. (E, F) Quantitative PCR analyses of SPP1 and MYH11 in the cerebral arteries of HU and HTN rats. (G, H) Cytoplasmic and mitochondrial Ca^2+^ levels in cerebral VSMCs of HU and HTN rats. *n* = 6. *p < 0.05, **p < 0.01, ***p < 0.001, ****p < 0.0001.

**FIGURE 2:**
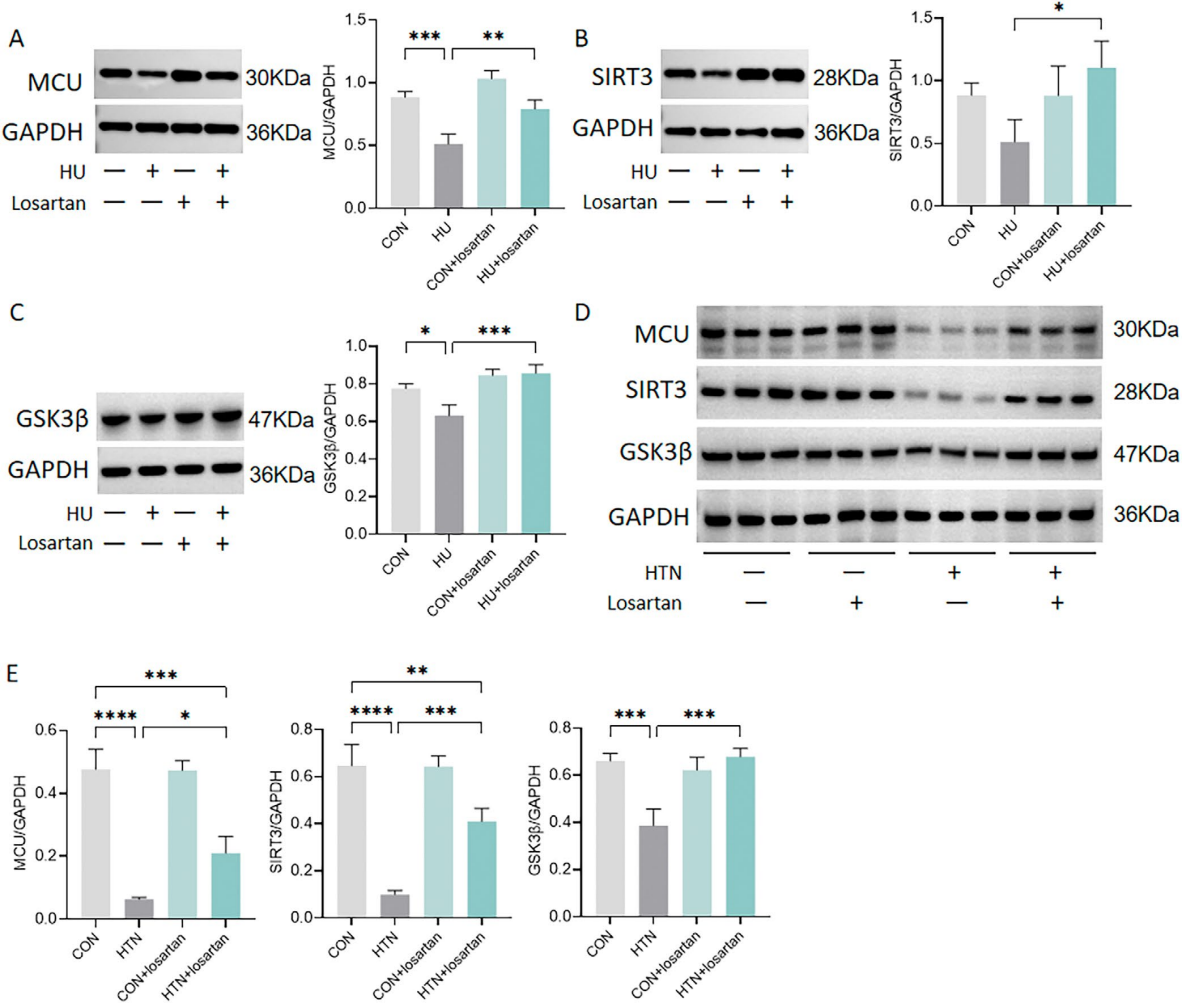
Expression of MCU, SIRT3, and GSK3β in the cerebral arteries of rats with hindlimb unloading (HU) and angiotensin II (Ang II)‐induced hypertension following Losartan administration. HU and hypertensive rats received distilled water containing losartan at 30 mg/kg/day or vehicle by gavage. (A, B) Western blot analyses of MCU and SIRT3 in HU rats, with or without Losartan administration. (C) Western blot analyses of MCU, SIRT3, and GSK3β in hypertensive rats, with or without Losartan administration. *n* = 6. *p < 0.05, **p < 0.01, ***p < 0.001, ****p < 0.0001.

To investigate the effects of HU on cerebrovascular MCU‐SIRT3‐GSK3β signaling, rats were treated with 21‐day HU or subjected to 21‐day Ang II‐induced hypertension. As shown in Figure 1A, the systolic blood pressure (SBP) and diastolic pressure (DBP) of the tail artery steadily increased throughout the 21‐day modeling period. Compared to control rats, the significantly reduced absolute mass of the left soleus in HU rats confirmed the reliability of the HU model (Table 2). Compared to controls, the expression of MCU and SIRT3 was significantly downregulated in HU rat cerebral arteries, with a more pronounced reduction observed in Ang II‐induced hypertensive rats (Figure 1B,C). Besides, the p‐GSK3β Ser9/total GSK3β ratio was elevated in both HU and HTN rats, whereas the expression of GSK3β was diminished in these animals, suggesting that GSK3β is suppressed in HU and HTN rats (Figure 1D). In HU and hypertensive rats, *Myh11* mRNA levels were significantly decreased, while *Spp1* mRNA levels were significantly increased compared to controls (Figure 1E,F). Our previous research suggested that the contractile markers α‐SMA, calponin, SM‐MHC, and caldesmon were decreased, while the synthetic markers OPN and elastin increased in cerebral arteries [5, 6]. Collectively, these results establish a consistent molecular signature of VSMC phenotypic transition from contractile to synthetic states in HU and hypertension models. Additionally, mitochondrial Ca^2+^ levels were reduced, while cytoplasmic Ca^2+^ levels were elevated in HU and hypertensive rats (Figure 1G,H). This is consistent with previous research showing increased cytoplasmic Ca^2+^ and decreased mitochondrial Ca^2+^ in cerebrovascular VSMCs of HU rats [7]. Furthermore, administration of losartan, a selective Ang II type 1 receptor antagonist, significantly reversed the decreased expression of MCU, SIRT3, and GSK3β in the cerebral arteries of HU rats (Figure 2A–C). And losartan also reversed the decreased expression of MCU, SIRT3, and GSK3β in hypertensive rats (Figure 2D,E). Therefore, we observed similar expression levels of MCU, SIRT3, and GSK3β, as well as comparable Ca^2+^ homeostasis in both HU and Ang II‐induced hypertensive rats, suggesting that RAS activation may serve as a crucial regulatory mechanism.

**TABLE 2:** Body mass, wet mass of the left soleus, and soleus‐to‐body mass ratio of control (CON) and hindlimb unloading (HU) rats.

Group	Body mass(g)		Wet mass of the left soleus	
	Initial	Final	Absolute(mg)	Relative, mg/g body mass
Control	236.1 ± 1.6	354.1 ± 2.5	141.2 ± 1.5	0.40 ± 0.005
Hindlimb	240.3 ± 1.8	346.8 ± 2.1	70.0 ± 1.6****	0.20 ± 0.003****

*Note:* Values are presented as means ± SEM. *n* = 24.

**** *p* < 0.0001.

### 3.2 The Effects of Ang II on MCU, SIRT3, GSK3β Expression in VSMCs In vitro

**FIGURE 3:**
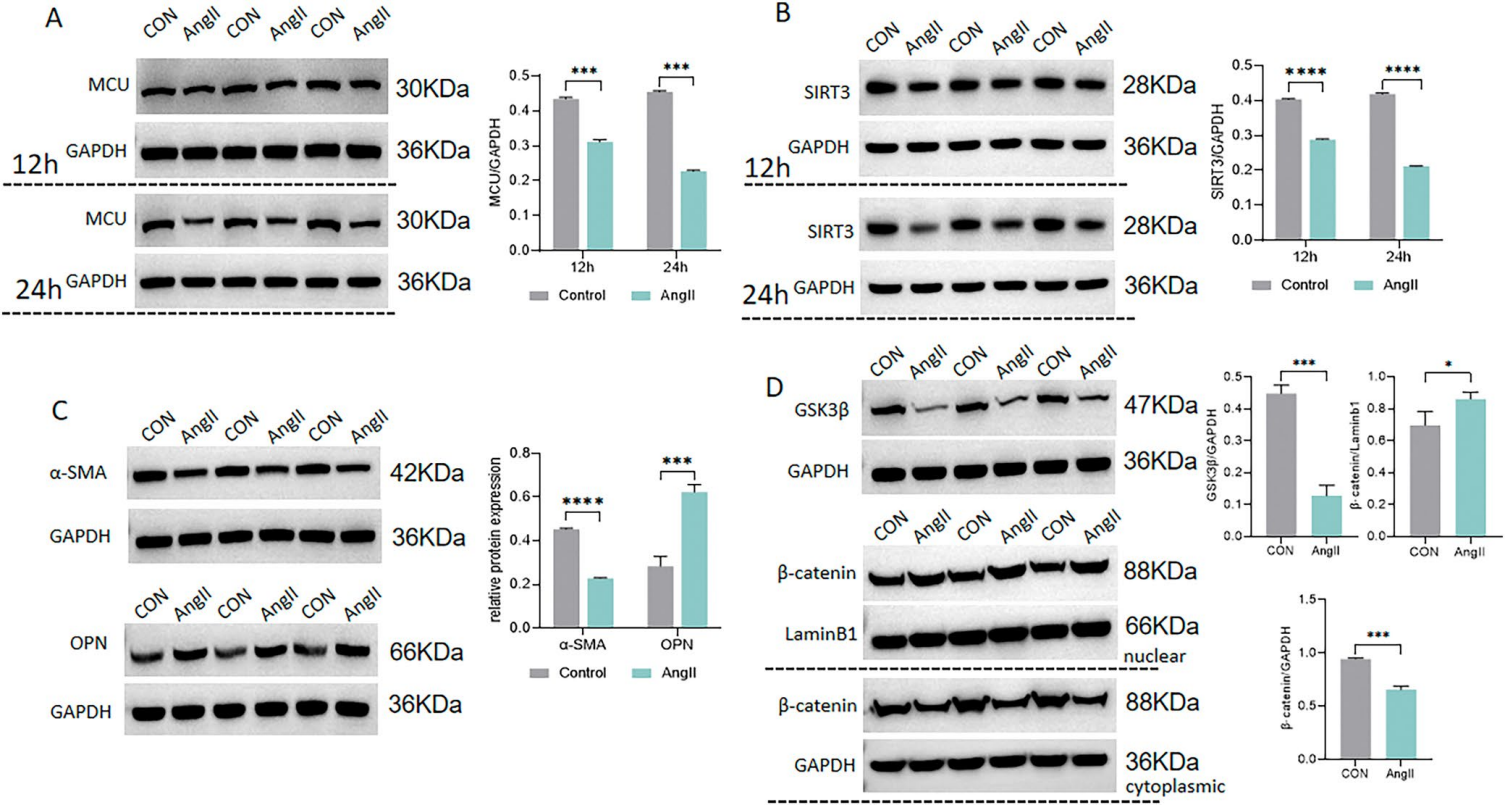
Expression of MCU, SIRT3, α‐SMA, OPN, GSK3β and β‐catenin in vascular smooth muscle cells (VSMCs) incubated In vitro with angiotensin II (Ang II). The VSMCs were incubated In vitro with Ang II (100 nmol/L). (A, B) Western blot analyses of MCU and SIRT3 in Ang II‐treated VSMCs after 12 and 24 h of incubation. (C) Western blot analyses of the contractile marker α‐SMA and synthetic marker OPN in Ang II‐treated VSMCs after 24 h of incubation. (D) Western blot analyses of nuclear and cytoplasmic β‐catenin and GSK3β in Ang II‐treated VSMCs after 24 h of incubation. *n* = 5. *p < 0.05, ***p < 0.001, ****p < 0.0001.

Given the activation of the RAS in the cerebral arteries of HU rats [4, 14], we sought to determine whether phenotypic switching of VSMCs under hindlimb unloading is associated with RAS activation and its underlying mechanism. To investigate this, VSMCs were incubated with Ang II In vitro. Compared to controls, the expression of MCU and SIRT3 was significantly reduced by Ang II, with a more pronounced reduction at 24 h compared to that at 12 h (Figure 3A,B). Additionally, Ang II decreased α‐SMA expression and increased OPN expression, indicating a synthetic transition of VSMCs following Ang II stimulation (Figure 3C). Previous studies have suggested that inhibiting GSK3β leads to the accumulation of β‐catenin, which in turn promotes phenotypic switching of VSMCs by regulating smooth muscle‐specific gene expression [14]. In this study, we demonstrated that GSK3β was inhibited in VSMCs incubated with Ang II (Figure 3D). Compared to controls, Ang II increased nuclear β‐catenin expression while decreasing cytoplasmic β‐catenin (Figure 3D). These results suggest that the inhibition of MCU and SIRT3 by Ang II is linked to the synthetic switching of VSMCs, which is potentially mediated by the GSK3β/β‐catenin pathway.

### 3.3 The Roles of MCU in Mitochondrial Redox Status and the Ubiquitin‐Mediated Degradation of SIRT3

**FIGURE 4:**
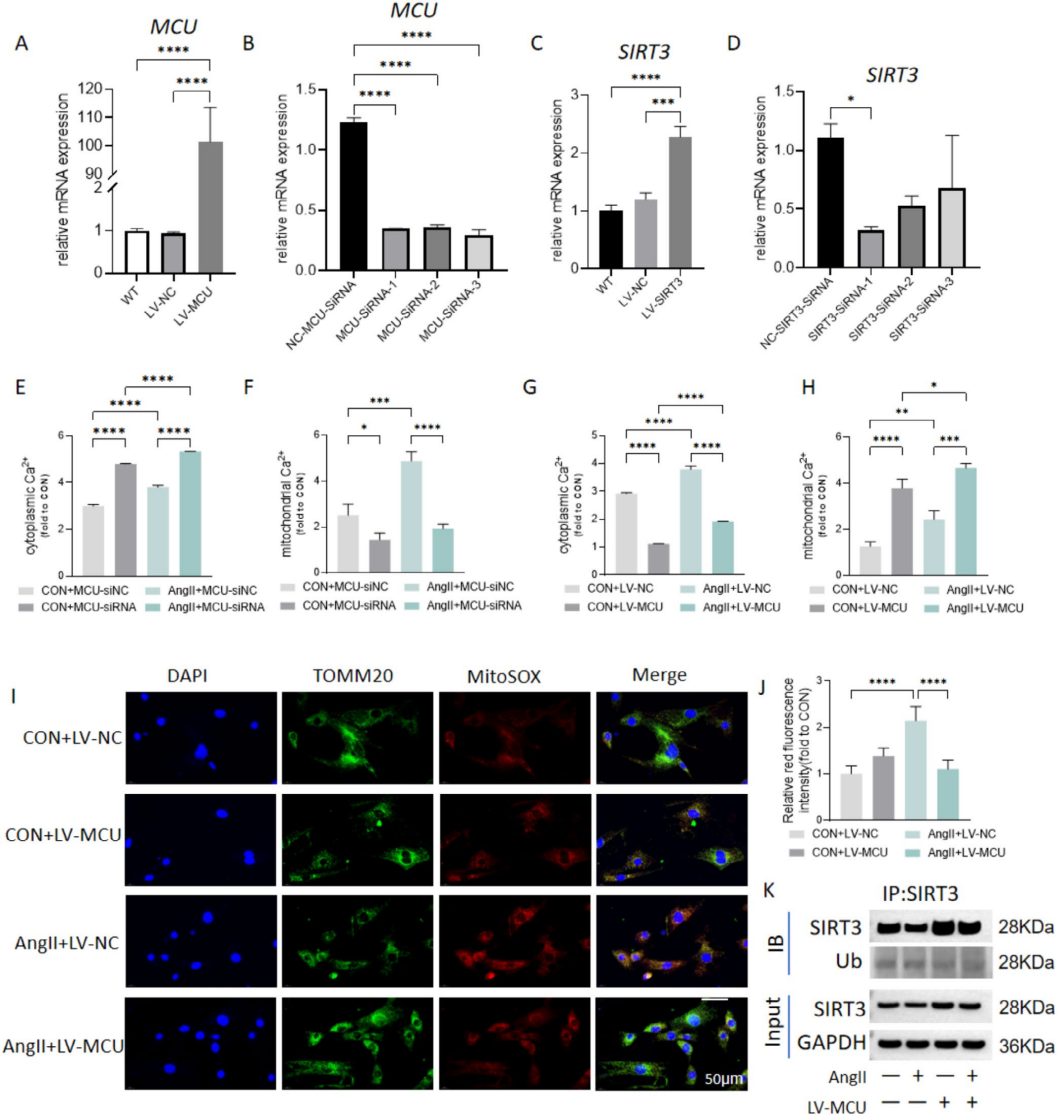
Disruption of Ca^2+^ buffering, mitochondrial oxidative stress, and SIRT3 ubiquitin‐mediated degradation following MCU inhibition in vascular smooth muscle cells (VSMCs). The VSMCs were incubated In vitro with angiotensin II (Ang II) (100 nmol/L). (A–D) Verification of overexpression lentivirus and knockdown plasmids targeting MCU and SIRT3. (E–H) Cytoplasmic and mitochondrial Ca^2+^ levels in VSMCs treated with Ang II for 24 h, following MCU overexpression and knockdown. (I, J) MitoSOX analysis of mitochondrial ROS in VSMCs treated with Ang II, with or without MCU overexpression. (K) Representative western blot images of VSMCs immunoprecipitated (IP) with SIRT3 and immunoblotted using ubiquitin (Ub) antibodies. Input SIRT3 is also shown. *n* = 5. *p < 0.05, **p < 0.01, ***p < 0.001, ****p < 0.0001.

To investigate the role of MCU in regulating calcium homeostasis and mitochondrial redox status, either MCU or SIRT3 was overexpressed or knocked down. As shown in Figure 4A–D, the efficiency of the plasmids was confirmed, with MCU‐siRNA3 and SIRT3‐siRNA1 being used in subsequent experiments. Compared to controls, incubation with Ang II significantly increased both cytoplasmic and mitochondrial Ca^2+^ levels (Figure 4E–H). Additionally, MCU knockdown led to an increase in cytoplasmic Ca^2+^ and a decrease in mitochondrial Ca^2+^ (Figure 4E,F). In the presence of Ang II, MCU knockdown further elevated cytoplasmic Ca^2+^ levels while decreasing mitochondrial Ca^2+^ (Figure 4E,F). Conversely, MCU overexpression reduced cytoplasmic Ca^2+^ levels while simultaneously increasing mitochondrial Ca^2+^ in both control and Ang II‐treated VSMCs (Figure 4G,H). Our previous research suggested that mtROS is involved in cerebrovascular remodeling [15], while it remains unknown whether MCU regulates mtROS. Furthermore, the production of Ang II‐induced mtROS was significantly reduced by MCU overexpression (Figure 4I,J). Co‐immunoprecipitation results showed that Ang II significantly increased the ubiquitination level of SIRT3, whereas MCU overexpression inhibited SIRT3 ubiquitination (Figure 4K). These findings suggest that inhibition of MCU disrupts Ca^2+^ homeostasis, promotes mtROS, and leads to the ubiquitination and degradation of SIRT3.

### 3.4 MCU Regulates GSK3β Acetylation Through SIRT3

**FIGURE 5:**
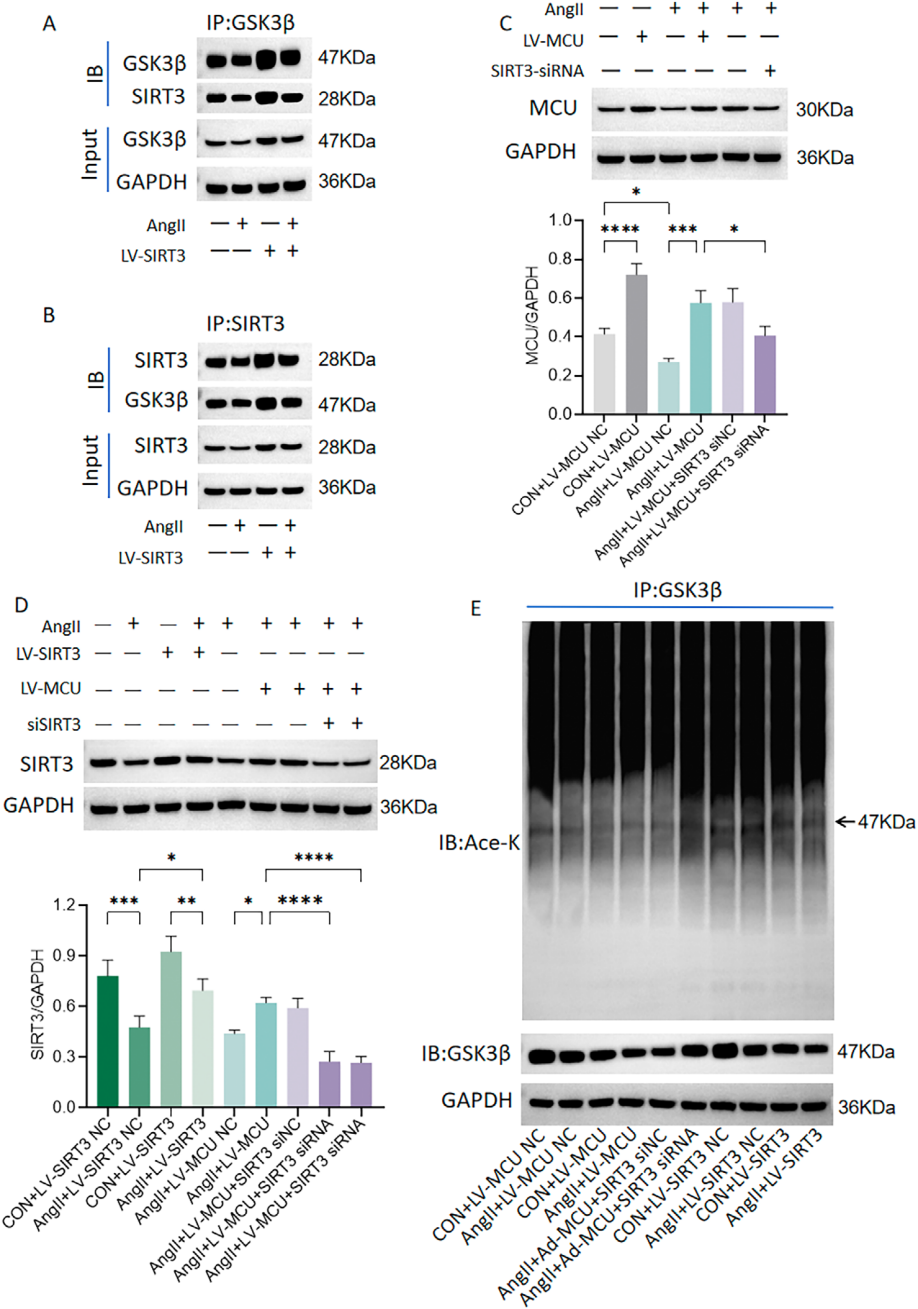
MCU regulates GSK3β acetylation via SIRT3 in vascular smooth muscle cells (VSMCs). The VSMCs were incubated In vitro with angiotensin II (Ang II) (100 nmol/L). (A) Representative western blot images of VSMCs immunoprecipitated with a GSK3β antibody and immunoblotted with GSK3β and SIRT3 antibodies. (B) Representative western blot images of VSMCs immunoprecipitated with a SIRT3 antibody and immunoblotted with GSK3β and SIRT3 antibodies. (C) Western blot analysis of MCU in VSMCs. (D) Western blot analysis of SIRT3 in VSMCs. (E) Representative western blot images of VSMCs immunoprecipitated with a GSK3β antibody and immunoblotted with anti‐acetyl‐lysine (Ace‐K) and GSK3β antibodies. *n* = 5. *p < 0.05, **p < 0.01, ***p < 0.001, ****p < 0.0001.

SIRT3 has been shown to regulate GSK3β acetylation, thereby modulating its activity [16, 17]. To investigate the mechanism through which SIRT3 regulates GSK3β, we first examined the endogenous interaction between SIRT3 and GSK3β. As shown in Figure 5A,B, co‐immunoprecipitation was performed using either anti‐GSK3β or anti‐SIRT3 antibodies, and the pull‐down samples were subsequently immunoblotted with SIRT3 and GSK3β antibodies, confirming the endogenous interaction between the two proteins in VSMCs. Compared to controls, Ang II reduced the interaction between SIRT3 and GSK3β, and this reduction was partially prevented by SIRT3 overexpression (Figure 5A,B). Furthermore, Ang II incubation decreased the protein expression of both MCU and SIRT3, but this effect was reversed by MCU overexpression (Figure 5C,D). The reversal of SIRT3 expression by MCU overexpression was further inhibited by SIRT3 knockdown (Figure 5D). Additionally, Ang II stimulation upregulated the expression of Ac‐GSK3β (acetylated GSK‐3β), which was reduced by MCU overexpression and further enhanced by SIRT3 knockdown (Figure 5E). Moreover, the increased acetylation of GSK3β induced by Ang II was also reversed by SIRT3 overexpression (Figure 5E). These findings suggest that MCU modulates GSK3β acetylation via SIRT3 regulation.

### 3.5 MCU‐ SIRT3‐ GSK3β/β‐Catenin Signaling Pathway Regulates Phenotypic Switching of VSMCs

**FIGURE 6:**
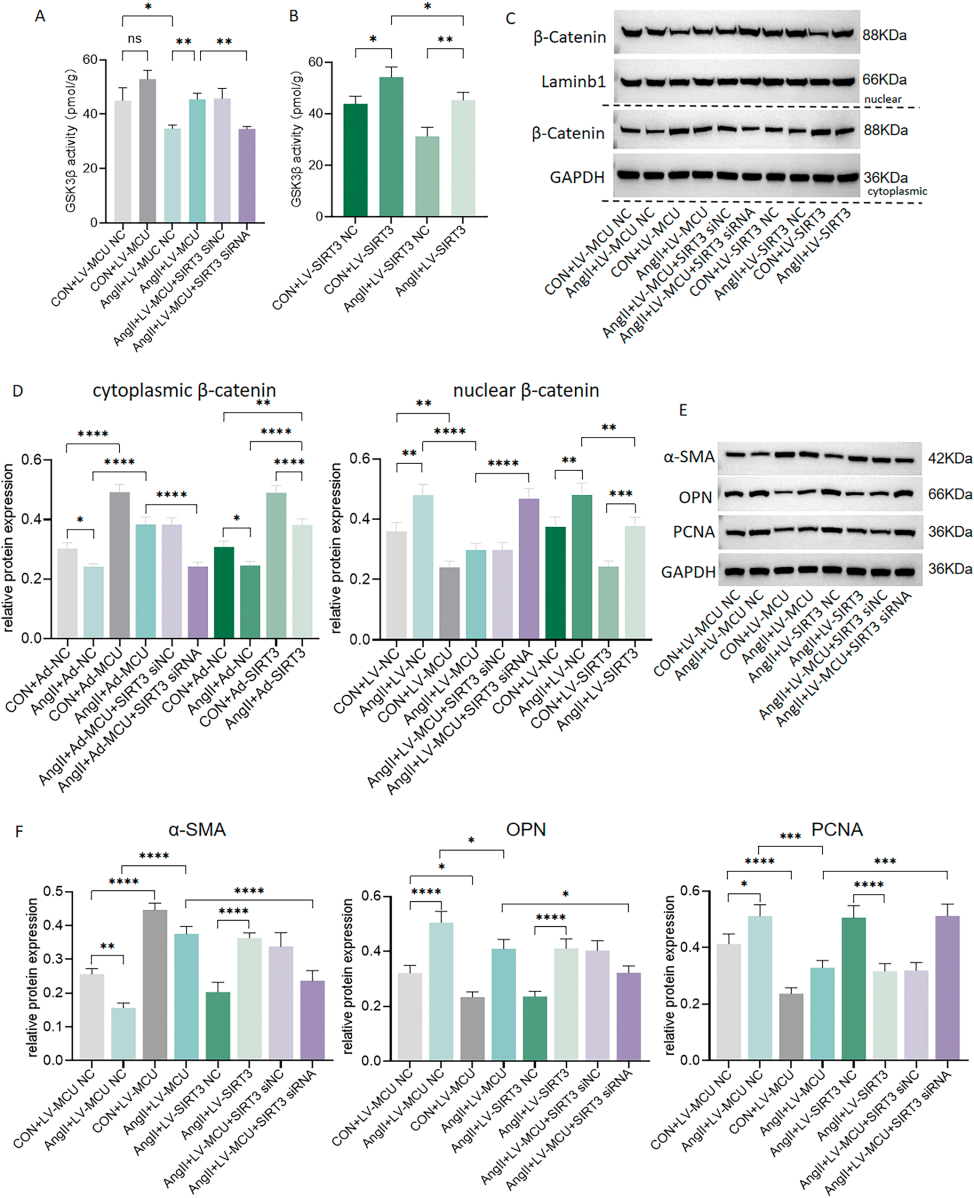
MCU regulates enzymatic activity of GSK3β and phenotype switching of vascular smooth muscle cells (VSMCs) via GSK3β/β‐catenin pathway. The VSMCs were incubated In vitro with angiotensin II (Ang II) (100 nmol/L). (A, B) Enzymatic activity of GSK3β in VSMCs. (C, D) Western blot analysis of nuclear and cytoplasmic β‐catenin in VSMCs. (E, F) Western blot analysis of α‐SMA, OPN and PCNA in VSMCs. *n* = 5. *p < 0.05, **p < 0.01, ***p < 0.001, ****p < 0.0001.

To determine the role of the GSK3β/β‐catenin signaling pathway in VSMC phenotypic switching, we examined GSK3β activity and cellular β‐catenin distribution. Compared to the control, Ang II significantly reduced GSK3β activity, which was enhanced by overexpression of SIRT3 and MCU (Figure 6A,B). Rescue experiments showed that the reversal of GSK3β activity following MCU overexpression was blocked by SIRT3 knockdown (Figure 6A). Regarding β‐catenin distribution, nuclear β‐catenin was increased by Ang II and then reduced by MCU overexpression, but further enhanced by SIRT3 knockdown (Figure 6C,D). Conversely, cytoplasmic β‐catenin exhibited an opposite trend (Figure 6C,D). When VSMCs transition from the contractile to the synthetic phenotype, they re‐enter the cell cycle and begin to proliferate. High PCNA expression is a key characteristic of synthetic phenotype VSMCs [18]. Additionally, α‐SMA was decreased by Ang II, but this effect was reversed by MCU overexpression and further reduced by SIRT3 knockdown. In contrast, OPN and PCNA showed the opposite pattern (Figure 6E,F). These results suggest that the SIRT3‐modulated GSK3β/β‐catenin signaling pathway plays a role in the phenotypic switching of VSMCs regulated by MCU.

### 3.6 MCU Regulates Vascular Remodeling Through SIRT3‐ GSK3/β‐Catenin Pathway

**FIGURE 7:**
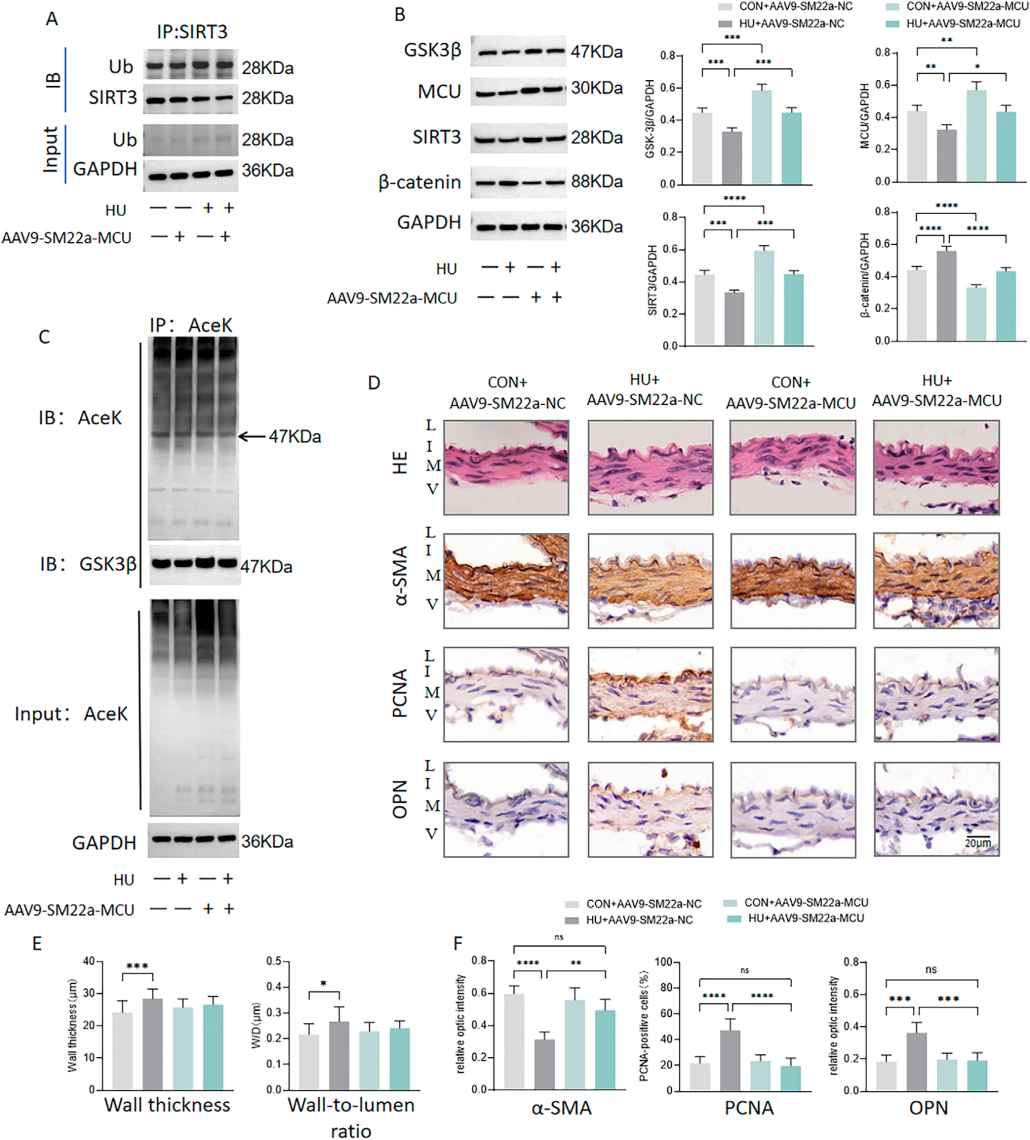
MCU regulates cerebrovascular remodeling in hindlimb unloading (HU) rats. Rats were subjected to 21‐day HU to simulate microgravity effects. (A) Representative Western blot images of cerebral arteries immunoprecipitated with a SIRT3 antibody and immunoblotted with ubiquitin (Ub) antibodies. (B) Western blot analysis of MCU, SIRT3, GSK3β, and β‐catenin in HU rat cerebral arteries. (C) Representative western blot images of cerebral arteries immunoprecipitated with a GSK3β antibody and immunoblotted with anti‐acetyl‐lysine (Ace‐K) antibodies. (D) Representative images of H&E staining and representative images of immunohistochemical staining for α‐SMA, OPN, and PCNA of basilar arteries in HU rats. (E) Quantitative analysis of wall thickness and wall‐to‐lumen ratio basilar arteries in HU rats. (F) Quantitative analysis of relative optical density of α‐SMA, OPN, and PCNA, calculated by normalizing the integrated optical density to vessel wall area. L, lumen; I, intima; M, media; and V, adventitia. *n* = 5. *p < 0.05, **p < 0.01, ***p < 0.001, ****p < 0.0001.

**FIGURE 8:**
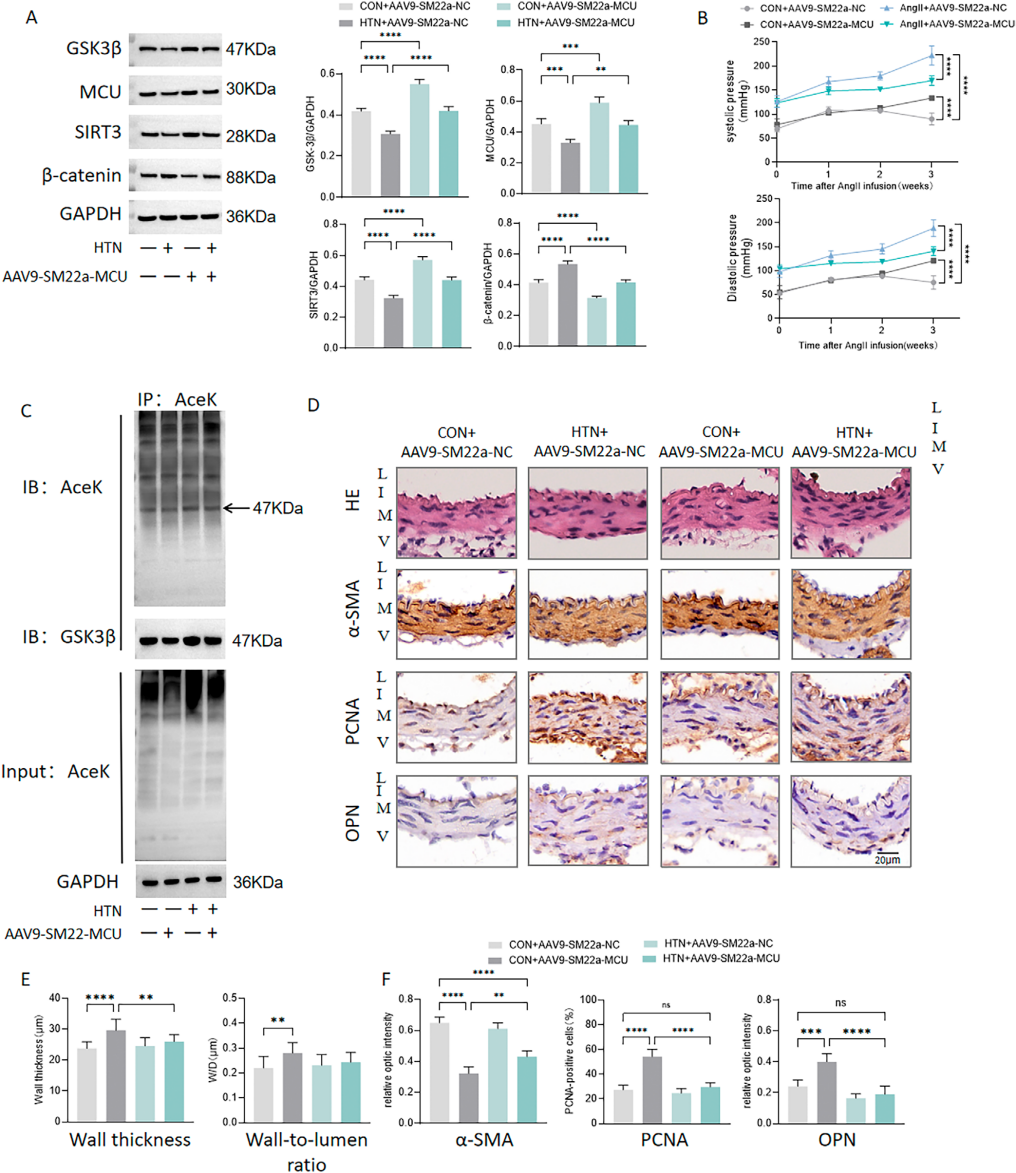
MCU regulates cerebrovascular remodeling in angiotensin II (Ang II)‐induced hypertensive rats (HTN). Rats were treated with a 21‐day Ang II infusion (0.7 mg/kg/day) to establish hypertension. (A) Noninvasive tail cuff monitoring of systolic and diastolic blood pressure in rats infused with Ang II. (B) Western blot analysis of MCU, SIRT3, GSK3β, and β‐catenin in cerebral arteries of hypertensive rats. (C) Representative Western blot images of cerebral arteries immunoprecipitated with a GSK3β antibody and immunoblotted with anti‐acetyl‐lysine (Ace‐K) antibodies. (D) Representative images of H&E staining and representative images of immunohistochemical staining for α‐SMA, OPN, and PCNA of basilar arteries in hypertensive rats. (E) Quantitative analysis of wall thickness and wall‐to‐lumen ratio basilar arteries in hypertensive rats. (F) Quantitative analysis of relative optical density of α‐SMA, OPN, and PCNA, calculated by normalizing the integrated optical density to vessel wall area. L, lumen; I, intima; M, media; and V, adventitia. *n* = 5. **p < 0.01, ***p < 0.001, ****p < 0.0001.

To investigate whether MCU regulates vascular remodeling through the mechanism described above, we overexpressed MCU in VSMCs and conducted studies in both HU rats and Ang II‐induced hypertensive rats. Compared to the control, the ubiquitination level of SIRT3 was significantly increased in HU rat cerebral arteries, which was partially reduced by MCU overexpression (Figure 7A). In comparison to the control, both HU and Ang II administration significantly decreased the protein expression of MCU, GSK3β, and SIRT3, while increasing β‐catenin expression (Figures 7B and 8A). Furthermore, MCU overexpression in VSMCs significantly reduced the increase in SBP and DBP of the tail artery in rats (Figure 8B). The acetylated GSK3β was elevated by HU and Ang II administration, but this effect was significantly reversed by MCU overexpression (Figures 7C and 8C). Furthermore, the wall thickness and wall‐to‐lumen ratio of the basilar arteries were increased in both HU and hypertensive rats (Figures 7D,E and 8D,E). Overexpression of MCU significantly reversed the increased wall thickness in hypertensive rats (Figure 8D,E). However, MCU overexpression did not protect against changes in wall thickness or wall‐to‐lumen ratio in the HU group, nor did it affect the wall‐to‐lumen ratio in the hypertensive group (Figures 7D,E and 8D,E). In terms of the phenotypic makers, α‐SMA was decreased, while OPN and PCNA levels were increased by HU and Ang II, effects that were subsequently reversed by MCU overexpression (Figures 7D,F and 8D,F). These results suggest that MCU regulates cerebrovascular remodeling through the GSK3β/β‐catenin pathway.

## 4 Discussion

To the best of our knowledge, this is the first study to demonstrate that Ang II downregulates MCU, leading to mitochondrial dysfunction and altering the phenotype of cerebral arterial VSMCs under conditions of hindlimb unloading and Ang II‐induced hypertension. The underlying mechanism involves enhanced ubiquitination of SIRT3, which subsequently modulates the downstream GSK3β/β‐catenin signaling pathway.

Mitochondria and the endoplasmic reticulum rely on Ca^2+^ for their function and play key roles in maintaining intracellular Ca^2+^ homeostasis. Mitochondria uptake large amounts of Ca^2+^ through an inner membrane transporter known as MCU. In humans, MCU complex consists of four main protein subunits: MCU, which forms the ion channel; EMRE, which is critical for MCU activity; and MICU1 and MICU2, which regulate its function [19]. The MCU complex has Ca^2+^‐selectivity at low cytoplasmic Ca^2+^ concentration, which allows mitochondrial uptake and release of Ca^2+^ to dynamically buffer cytoplasmic Ca^2+^. Mitochondrial Ca^2+^ regulates various enzymes involved in metabolic processes, affects ATP synthesis, modulates Ca^2+^ signaling, and influences both apoptosis and autophagy [20, 21]. In the present study, we first observed a reduction in MCU levels in the cerebral VSMCs of both HU rats and Ang II‐induced hypertensive rats. Meanwhile, both our previous research and the data from the present study suggest that HU and hypertension induce a switch to a synthetic phenotype in VSMCs, characterized by increased expression of OPN and elastin, along with decreased levels of α‐SMA and SMMHC [5, 6]. Enhanced NFAT expression and CREB phosphorylation have been reported in HU and hypertensive rats [5, 22, 23]. An increase in cytoplasmic Ca^2+^ activates the Ca^2+^‐dependent transcription factor NFAT, which subsequently upregulates CREB and induces its phosphorylation [20]. This process triggers excitation‐transcription coupling in VSMCs, promoting the transcription of proliferative genes and driving cell proliferation. However, the relationship between MCU and VSMC phenotype seems to vary depending on the specific pathophysiological conditions. In lymphoblastoid cell lines derived from hypertensive individuals with the tRNA (Ile) A4263G mutation, MCU levels are significantly lower compared to those in normotensive individuals [24]. In ApoE^−/−^ mice, increased MCU expression promotes VSMC proliferation and the formation of atherosclerotic plaques [8]. This may result from distinct MCU‐dependent signaling cascades, particularly those involving oxidative stress and cellular Ca^2+^ distribution.

The effects of MCU on the cardiovascular system may be twofold. Following myocardial ischemia/reperfusion, excessive activation of MCU leads to mitochondrial Ca^2+^ overload, inhibition of ATP production, disruption of the mitochondrial membrane potential, and opening of the permeability transition pore, ultimately triggering apoptosis [25]. MCU depletion exacerbates isoproterenol‐induced cardiac hypertrophy, fibrosis, contractile dysfunction, and cardiomyocyte death [26]. In contrast, cardiac‐specific overexpression of MCU maintains Ca^2+^ homeostasis, preserves contractile function, and prevents cardiac hypertrophy [26]. These findings suggest that proper MCU expression levels and mitochondrial Ca^2+^ uptake capacity are crucial for maintaining normal cellular function. Both decreased and excessive MCU expression leads to cellular dysfunction, underscoring the importance of maintaining optimal MCU activity for cellular homeostasis. In this study, we observed a reduction in MCU expression and mitochondrial Ca^2+^ levels in both HU and Ang II‐induced hypertensive rat models, which was closely associated with the synthetic phenotypic switching of VSMCs. These findings align with previous reports in models of pulmonary hypertension and spontaneous hypertension [9, 24]. However, we observed that overexpression of MCU in VSMCs of control rats resulted in elevated tail artery blood pressure during the third week, which surpassed both the normal range (85–115 mmHg) and the blood pressure of control rats. Previous studies suggest that the elevation of MCU‐induced Ca^2+^ levels also promotes the generation of mROS [27], a phenomenon that has been demonstrated in PASMCs [28]. Based on these findings, we speculate that excessively high MCU expression levels could also lead to pathological vascular changes. Nevertheless, determining the optimal MCU level remains a challenging issue that warrants further investigation.

Recent studies have shown that RAS regulates the expression of MCU in cardiomyocytes; however, it remains unclear whether this holds true in VSMCs. MCU expression is upregulated after Ang II stimulation, which is associated with mitochondrial Ca^2+^ overload and pathological remodeling of the left ventricle [10]. In the present study, the decreased MCU expression in cerebral arteries of HU and hypertensive rats was associated with increased cytoplasmic but decreased mitochondrial Ca^2+^ levels. In Ang II‐induced VSMCs, the expression of MCU is reduced, whereas the cytoplasmic and mitochondrial Ca^2+^ levels in Ang‐II‐incubated VSMCs are elevated. This may be due to an increase in the total intracellular Ca^2+^ content, as Ang II can activate L‐type voltage‐gated Ca^2+^ channels and affect the sodium‐calcium exchanger [29]. The discrepancies may stem from differences in the concentration of Ang II stimulation and the cell types employed. VSMCs regulate contractile function primarily through the redistribution or influx of intracellular Ca^2+^, rather than by increasing mitochondrial Ca^2+^ [30]. Furthermore, VSMCs are prone to phenotypic switching in response to Ang II stimulation. During this process, VSMCs must adjust their intracellular metabolism and energy demands, and a reduction in mitochondrial Ca^2+^ may serve as a strategy for adapting to this functional remodeling [9, 31]. The mitochondrial MCU can take up Ca^2+^ from the endoplasmic reticulum via IP_3_R, while Ang II induces a reduction in IP_3_R expression in VSMCs [32]. Recent studies have shown that Ang II can also increase MICU1 expression, thereby raising the threshold for mitochondrial Ca^2+^ uptake [33]. Therefore, the roles of the RAS in regulating MCU expression in cardiomyocytes and VSMCs may differ, and further evidence is needed to clarify the underlying mechanisms.

SIRT3 is a mitochondrial deacetylase that plays a crucial role in maintaining mitochondrial stability and function, including regulating mitochondrial metabolic reprogramming, the oxidative stress response, membrane potential maintenance, ATP production, and mitophagy. In the present study, we observed a reduction in SIRT3 expression in the cerebral arteries of HU rats. We have previously shown that oxidative stress is increased in the cerebral arteries of HU rats, suggesting an association between reduced SIRT3 and the activation of oxidative stress [4]. Under stress conditions, SIRT3 can be degraded via the ubiquitin‐proteasome system [34]. In the present study, we observed an increased level of SIRT3 ubiquitination in Ang II‐induced VSMCs and in the cerebral arteries of HU rats, which was associated with heightened mtROS. Notably, these effects were mitigated by MCU overexpression. Ang II stimulation can lead to increased mtROS, while overexpression of MCU alleviates mtROS and the ubiquitination‐mediated degradation of SIRT3. This suggests that impaired mitochondrial Ca^2+^ buffering caused by Ang II affects mitochondrial oxidative stress status, thereby regulating SIRT3 protein stability. Previous studies have shown that the absence of SIRT3 results in vascular dysfunction and hypertension [35]. These results reveal a novel mechanism by which MCU regulates SIRT3 stability.

In this study, we are the first to demonstrate that SIRT3 regulates phenotypic switching of VSMCs and vascular remodeling by targeting GSK3β in HU and Ang II‐induced hypertensive rats. Previous studies have shown that Ang II promotes the phosphorylation of GSK3β at the Ser9 site, thereby inhibiting its activity [36]. Building on this, we discovered a novel acetylation‐based regulatory mechanism in which Ang II suppresses GSK3β activity through acetylation. This acetylation negatively regulates GSK3β's enzymatic activity by impairing its ability to phosphorylate downstream substrates. GSK3β is a constitutively active kinase involved in key cellular functions such as growth, survival, apoptosis, and vascular aging [37]. Notably, we found that SIRT3 directly interacts with GSK3β, deacetylates it, and enhances its enzymatic activity. This deacetylation prevents β‐catenin from promoting the expression of proliferative and synthetic genes, which play a central role in regulating vascular remodeling [38]. Furthermore, we observed a significant reduction in mtROS in VSMCs following the overexpression of both SIRT3 and MCU. This suggests that SIRT3 may exert protective effects in both HU and hypertensive rats, primarily through the SIRT3‐GSK3β deacetylation pathway and its associated antioxidant mechanisms. These findings provide novel insights into the molecular mechanisms by which SIRT3 modulates vascular remodeling and cellular responses to stress in the context of hypertension and vascular injury.

The limitations still exist. First, as MCU complex consists of multiple subunits, it remains unclear whether other key regulatory proteins are involved in vascular remodeling. Second, previous studies have demonstrated that SIRT3 can directly regulate the acetylation level of β‐catenin [39], thereby affecting its protein activity. We cannot rule out the possibility that SIRT3 may also directly influence β‐catenin in vascular remodeling. Our ongoing research into the specific lysine site through which SIRT3 regulates GSK3β may provide further insights. Third, the animal model only includes male rats, thus overlooking the potential impact of gender differences in vascular biology, which should be addressed in future studies.

## 5 Conclusion

We propose a novel mechanism in which, in HU and hypertensive rats, the downregulation of MCU disrupts intracellular calcium homeostasis and promotes mtROS. This oxidative stress triggers the ubiquitin‐mediated degradation of SIRT3, which in turn inhibits GSK3β and facilitates the nuclear translocation of β‐catenin. Subsequently, this stimulates the expression of proliferative and synthetic genes in VSMCs, contributing to vascular remodeling. Therefore, targeting MCU represents a promising therapeutic strategy for vascular remodeling in the contexts of microgravity and hypertension.

## Author Contributions

Ran Zhang and Min Jiang conceived and designed the experiments. Min Jiang, Lejian Lin, Shuai Yue, Shujin Shi, and Haojie Yan performed the experiments. Hui Yi, Fan Han, Shuai Xu, and Junjie Su performed data analysis. Ran Zhang, Min Jiang, and Lejian Lin wrote the manuscript. All the authors have full access to all the data in the current study and final responsibility for the decision to submit for publication.

## Conflicts of Interest

The authors declare no conflicts of interest.

## Data Availability

The data that support the findings of this study are available from the corresponding author on reasonable request.
